# Antioxidant Activity in Bee Products: A Review

**DOI:** 10.3390/antiox10010071

**Published:** 2021-01-07

**Authors:** Marianna Martinello, Franco Mutinelli

**Affiliations:** Istituto Zooprofilattico Sperimentale delle Venezie, NRL for Honey Bee Health, 35020 Legnaro, Italy; fmutinelli@izsvenezie.it

**Keywords:** beeswax, honey, propolis, pollen, royal jelly, venom, antioxidant, polyphenols, honeybee, stingless bee

## Abstract

Bee products have been used since ancient times both for their nutritional value and for a broad spectrum of therapeutic purposes. They are deemed to be a potential source of natural antioxidants that can counteract the effects of oxidative stress underlying the pathogenesis of many diseases. In view of the growing interest in using bioactive substances from natural sources to promote health and reduce the risk of developing certain illnesses, this review aims to update the current state of knowledge on the antioxidant capacity of bee products such as honey, pollen, propolis, beeswax, royal jelly and bee venom, and on the analytical methods used. The complex, variable composition of these products and the multitude of analytical methods used to study their antioxidant activities are responsible for the wide range of results reported by a plethora of available studies. This suggests the need to establish standardized methods to more efficiently evaluate the intrinsic antioxidant characteristics of these products and make the data obtained more comparable.

## 1. Introduction

Since ancient times, beekeeping products as honey, propolis, pollen, royal jelly, beeswax and bee venom have been among the most commonly used natural products in folk medicine by virtue of their powerful healing properties and high bioactive molecule content [1]. This branch of traditional medicine, with its scientific foundations, is now called apitherapy and is used to prevent or heal a number of different conditions, as wounds, rheumatic diseases, immune and neurologic conditions, and alimentary tract disorders, among others [2,3]. Nowadays, diet and a balanced life style are widely acknowledged to play an important role in the prevention and treatment of diseases. To improve their quality of life, modern consumers increasingly seek and use so-called natural functional foods containing bioactive substances of natural origin, thanks in part to their greater safety compared to synthetic drugs [4,5]. Scientific studies attribute to bee products a broad range of beneficial health effects, including antioxidant, antibacterial, anti-inflammatory, antitumor, antiviral properties, and many others [6,7]. One of the most important properties is their antioxidant capacity, which contributes to the prevention of certain illnesses, protecting cells against damage by oxidative agents such as free radicals. These are highly unstable, and therefore very reactive atoms, molecules or compounds due to their atomic or molecular structure, which has one or more unpaired electrons. They attempt to pair up with other molecules, atoms, or even individual electrons to create a stable compound, receiving electrons from other atoms. This generates reactive oxygen species (ROS), and free radicals bring about molecular transformations and gene mutations in many types of organisms. This is called oxidative stress and is deemed to contribute to the development of chronic and degenerative diseases such as cancer, autoimmune disorders, aging, cataracts, rheumatoid arthritis, and cardiovascular and neurodegenerative diseases [8]. ROS are produced naturally by metabolism or result from poor living conditions and environmental pollution [9]. The radical theory in human physiology claims that active free radicals are involved in almost all cellular degradation processes and lead to cell death. Antioxidants are molecules capable of slowing or inhibiting the oxidation of other molecules, thereby preventing such changes. Plant antioxidants display great bioactivity and molecular diversity and are present in honey and other bee products [10]. For example, since honey is produced by bees from nectar or plant secretions, various substances are transferred from plants and accumulated in this food. Consequently, the composition of honey, including its physical, chemical, organoleptic, and nutraceutical properties, is directly linked to the geographical, climatic and environmental characteristics of the areas where it is produced [11]. These differences represent a useful discriminatory tool for the classification and identification of honey. 

The *Apis mellifera* L. honeybee currently dominates the world beekeeping market. However, this review considers another bee species, belonging to the family Hymenoptera and subfamily Meliponinae. They are known as “stingless bees” because their sting is greatly reduced and not used for defense purposes; rather, they defend their nest by biting [12,13]. They play a significant role in plant pollination, pollinating an estimated 40–90% of native or cultivated species in the tropics [13]. The Meliponini tribe consists of over 600 species (61 genera) distributed in tropical zones worldwide, mainly the Neotropics, South and Central America, tropical Africa, Southwest Asia, and Australia [13,14]. Stingless bees produce and store much less honey on a per hive basis (1–5 kg) than do *A. mellifera* bees, which are the world leader in honey production, with an average of 20 kg of honey per hive. For this reason, their honey is less well known, quality control standards are absent, and industrial production levels are low [12]. Stingless bee honey has gained attention in recent years owing to its distinctive characteristics, as its exotic flavor, and being more exposed to propolis, to its higher probability of becoming infused with plant-derived antimicrobial compounds compared to *A. mellifera* honey [15].

Our review on the antioxidant activity (AOA) of bee products focuses on the most recently published papers, from approximately 2010 to the present date, given the extent of the topic. We consider in more detail the “non-biological” AOA tests that use reagents to trigger a colorimetric reaction in the presence of antioxidant substances, allowing them to be determined qualitatively and quantitatively. We decided to focus on these chemical assays as they are more standardized, currently used for the determination of AOA and supported by the relevant literature. In vitro and in vivo studies are seldom used to quantify the AOA of bee products, with a wide variability in tests and results, which we will outline below. The antioxidant properties of bee products have been applied to biological systems in vitro and in vivo, e.g., on cell lines [16,17,18,19,20,21,22,23,24,25,26,27,28,29], blood cells [30,31,32,33,34], or in different types of diseases in animal models and humans [3,35].

## 2. Antioxidant Compounds in Bee Products

Plant antioxidants are synthesized by plants to counteract biotic (pathogenic, predatory, competitive species) and abiotic (UV radiation, desiccation, thermal shock) stresses and favor the attraction of pollinators, the dispersion of seeds and allelopathic phenomena [9]. Secondarily, they affect the health of people who consume them through food, including honeybee products produced by bees from floral nectar, pollen, or plant secretions. Plant antioxidants are highly bioactive and present great molecular diversity, but phenolics (phenolic acids, flavonoids) are the most abundant and have the highest antiradical activity [36]. Phenolic compounds range from simple, low molecular-weight, single aromatic-ringed compounds to large, complex tannins and derived polyphenols [37]. Phenolic acids can be divided by chemical structure into hydroxybenzoic acids, with a C1–C6 nuclear structure derived from benzoic acid, with different methylation and hydroxylation of the aromatic ring (e.g., gallic acid, benzoic acid and vanillic acid); and hydroxycinnamic acids, with a C3–C6 general structure and differences in the originating ring substituents (e.g., caffeic acid, p-coumaric acid, ferulic acid, and cinnamic acid). Flavonoids have a C6–C3–C6 general structure, linking two benzene rings connected by a pyran ring, and can be classified into flavones, flavanones, and flavonols according to the type of substituent present on the ring (e.g., catechin, myricetin, quercetin, apigenin, kaempferol, luteolin, rutin, isorhamnetin, pinocembrin, or gallochatechin) [38]. The consulted literature refers only to active substances present in bee products and does not consider possible metabolites derived from honeybee metabolism. Furthermore, the assays used to determine the antioxidant compounds and AOA identify groups of compounds with similar chemical properties and not the single active substances. Different mechanisms underlie the antioxidant capacity of phenols, such as free-radical scavenging, donation of hydrogen, metal ion chelation, single oxygen quenching, and action as a substrate for superoxide and hydroxyl radicals [39,40]. These mechanisms are strictly linked to the metabolites and their molecular structure, e.g., the readiness of hydrogen donation may be affected by the steric hindrance of the carboxyl group, located next to the hydroxyl group. This indicates that the number and position of hydroxyl groups in phenolic compounds are paramount to the scavenging capacity of antioxidants [41]. AOA is highly correlated with phenolic compounds, but bee products are multicomponent natural substances and therefore also contain other substances presenting AOA, including minerals, amino acids, peptides, proteins, organic acids, and enzymes, but at lower concentrations than phenols [38]. Type and concentration are primarily influenced by the bee product in question, followed by botanical source, geographical and entomological origin, and climatic conditions [35].

## 3. Determination of Antioxidant Compounds and Activity 

A broad array of assays is available to quantify phenolic content and AOA within plant extracts and pure compounds. There is no official method for AOA determination and none of the methods used are ideal, each being designed to measure a different group of antioxidants. Different methods can yield different results since oxidation is a complex process occurring in several stages in vivo and AOA can be measured by different mechanisms. For example, some methods are based on the electron or hydrogen transfer reaction, others are designed to evaluate the ability to inhibit the formation of ROS or the chelation of metal ions. Given the complexity of in vivo antioxidant action mechanisms, and the complex interactions between intrinsic and extrinsic factors present in biological matrices, several laboratory assays have been developed and are often performed on the same sample to more closely investigate the antioxidant potential of natural products. Antioxidants can respond differently to different radical or oxidant sources, and no single assay will accurately reflect all radical sources or all antioxidants in a mixed or complex system. Table 1 summarizes the methods of quantifying the bioactive molecules and AOA of the beehive products most commonly encountered in drawing up this review [42,43,44,45,46,47,48,49,50,51,52,53,54,55,56,57,58,59,60,61,62,63,64,65,66,67,68].

Plant polyphenols can act as reducing agents, hydrogen atom donators, singlet oxygen scavengers, or transition-metal ion chelators [53]. In general, according to the reaction mechanisms, methods can be divided into single electron transfer (SET) and hydrogen atom transfer (HAT) methods. SET methods detect the ability of an antioxidant substance to transfer an electron to reduce a compound, including radicals, carbonyls, and metals. Relative reactivity in SET methods is based primarily on deprotonation and the ionization potential of the reactive functional group, hence SET reactions are pH dependent [67]. SET methods include total phenolic content (TPC) determination, the 2,2-diphenyl-1-picrylhydrazyl (DPPH) free-radical scavenging assay, and the ferric reducing/antioxidant power (FRAP) assay, among others. HAT methods measure the potential of an antioxidant to quench free radicals by hydrogen donation. HAT reactions are solvent, pH independent, and usually fast [67]. The presence of reducing agents, including metals, is a complication in HAT assays and can lead to erroneously high apparent reactivity [69]. These methods include ORAC and β-carotene bleaching assays. Other methods reflect, for example, the scavenging potential of different radicals, such as superoxide, hydroxyl, or hydrogen peroxide radicals. The detection methods most commonly used to evaluate the antioxidant properties of bee products are listed in Table 1. We endeavored to trace the original methods most frequently cited by the studies considered in our review, which explains why some references date back much further than the last ten years on which we sought to focus.

Results are often expressed as equivalents, calculated through calibration curves using standard antioxidant substances, such as gallic acid (GAE) for the TPC assay; catechin (CAE), quercetin (QE), and rutin (RE) for TFC determination; Trolox (6-hydroxy-2,5,7,8-tetramethylchroman-2-carboxylic acid, TE), a water-soluble analog of vitamin E, and ascorbic acid (AAE) for DPPH, ABTS, ORAC, and CUPRAC assays; and FeSO_4_*7H_2_0 (Fe^2+^) for FRAP.

AOA can be expressed as a percentage of radical scavenging activity (%RSA), calculated as follows:

%RSA = [(B−A/B)] × 100(1)

where B means “before” absorbance and refers to absorbance from the reagents without adding the sample (blank), and A means “after” absorbance, which refers to absorbance of the sample after the reaction.

Another way to express the result is by the extract concentration providing 50% of radical scavenging activity (e.g., the concentration of honey sample needed to scavenge 50% of DPPH), defined as EC50. The % RSA is plotted against the sample concentration and the EC50 value is calculated from the graph by linear regression analysis. The lower the EC50 value, the higher the scavenging capacity of the sample, as a less radical scavenger is required to reduce, say, DPPH.

Even when investigators use the same method, modifications are often incorporated (e.g., the extraction solvent, volume or procedure, incubation time, reference standard, etc.), making it hard to compare the results of the same test performed by different laboratories.

## 4. Honey

Council Directive 2001/110/EC relating to honey [70] stipulates that honey is “the natural sweet substance produced by *A. mellifera* bees from the nectar of plants or secretions of living parts of plants or excretions of plant-sucking insects on the living parts of plants, which the bees collect, transform by combining with specific substances of their own, deposit, dehydrate, store and leave in honeycombs to ripen and mature” (Annex I, point 1). Honey has been used by humans as a medicinal remedy since ancient times [71], from ancient Egyptian and classical civilizations (Greeks and Romans), who used it in medicinal or cosmetic formulations or as an embalming substance, to Arab peoples in the Middle Ages, for whom honey was the basis of pharmacy, as reflected in the Koran. Later, with the advent of antibiotics and other drugs, the use of honey for curative purposes was abandoned, mainly due to the absence of evidence-based studies. In recent decades, however, several investigations have demonstrated and explained the bioactive properties for which honey was empirically used [43]. One therapeutic property is the presence, among its various components, of natural antioxidants. 

Honey contains about 200 compounds, consisting mainly of sugars (fructose 25–45% and glucose 20–40%), water, and other substances, such as amino acids, enzymes, protein, vitamins, minerals, ash, organic acids, and phenolic and flavonoid compounds, which greatly contribute to its biological activity [72]. The therapeutic potential of honey is associated with the presence, variety, and quantities of bioactive compounds. This in turn depends on the type of flora, geographical location of production, climatic conditions, seasonal factors, soil composition, as well as the production process [73,74]. Consideration should also be given to the influence that analytical methods, such as sample dilution and chromatographic conditions, can have on this type of analysis. The vast majority of bioactive compounds in honey consist of molecules with phenolic structures, such as phenolic acids, flavonoids, procyanidins, and anthocyanins, vitamin C (ascorbic acid), vitamin E, carotenoids, enzymes (e.g., catalase, peroxidase), Maillard reaction products, and trace elements [48,75]. The basic composition of phenolic compounds in different varieties of honey is relatively similar and includes phenolic acids, such as caffeic, ellagic, ferulic, and p-coumaric acids; flavonoids, including apigenin, chrysin, galangin, hesperetin, kaempferol, pinocembrin, and quercetin; and antioxidants, such as tocopherols, ascorbic acid, superoxide dismutase (SOD), catalase (CAT), and reduced glutathione (GSH) [76]. Nonetheless, honey can contain some variety-specific compounds which can be used as markers of botanical origin [77]. 

Table 2 summarizes the results of the most recent publications on honey AOA [6,9,14,19,36,39,43,48,55,60,72,74,75,77,78,79,80,81,82,83,84,85,86,87,88,89,90,91,92,93,94,95,96,97,98,99,100,101,102,103,104,105,106,107,108,109,110,111,112,113,114,115,116,117]. The first general observation is to confirm the well-known positive correlation between phenolic content, color, and AOA of honey. This has been clearly demonstrated by Al-Farsi et al. [78], Can et al. [36], and Kuś et al. [99], among others, who spectrophotometrically assessed the color intensity of honey. Honey color depends on nectar source, pollen content, phenolic compounds, ash, minerals, and Maillard reaction products. Generally, dark-colored honeys have been reported to possess high levels of these substances and, for example, buckwheat and heather honey are characteristically dark brown, almost black in color. These varieties of honey are often the richest sources of antioxidants, and can reduce ROS-induced oxidative stress [19,90,99]. There is usually a correlation between TPC, TFC, and AOA, but similar TPC and TFC content does not always correspond to similar antioxidant capacity [43,78]. This is because the overall antioxidant capacity of each sample results from the combined activity of other nonphenolic compounds, although phenols do remain the largest class of antioxidants found in nature [114]. 

Honey composition and bioactivity were found to depend mainly on floral source, but external factors, such as geographical, seasonal, and environmental conditions, also play a role [84]. Comparing honey of the same botanical source but different geographical origin, Karabagias et al. [96] and Silici et al. [111] (located in Greece and Turkey, respectively) found TPC and AOA to be significantly affected by region. By contrast, Bodó et al. [84] reported that none of the antioxidant parameters were influenced by the Hungarian region of origin of their samples. As regards seasonality, Bartkiene et al. [6], for example, compared honey collected in spring and summer, observing that, on average, spring honeys had 25% less TPC.

Another aspect studied in various works is the influence of the extraction solvent on the antioxidant power of honey. Lianda et al. [101] and Rodríguez et al. [108] evaluated TPC and AOA in both crude honey and its methanol extract, generally obtaining lower TPC content in extracts, but finding a different pattern for AOA. It is well known that minerals, especially iron, can complex with phenolic compounds, enhancing their AOA. The water cleansing process applied during extraction can remove most of the minerals in honey, as their complexes are mainly water soluble. In addition, glycosylated phenolic compounds may not be extracted in the methanolic phase or may be lost in the aqueous phase [108]. Accordingly, there could be considerable changes in AOA and phenolic compounds when comparing honey and its extract. In general, according to the studies evaluated, honey dissolved in water yields higher polyphenol values, while extraction with methanol results in higher flavonoid levels [55,101].

There is a growing body of scientific research supporting the therapeutic potential of Manuka honey. It is a dark, monofloral honey produced by honeybees foraging on the Manuka tree (*Leptospermum scoparium*) native to New Zealand and Australia, and is classified and sold according to its methylglyoxal concentration or “Unique Manuka Factor” (UMF) [103]. Manuka honey is known for its excellent AOA and antibacterial activity [17] and has consequently been used for comparison with honeys of different botanical origin in several works. Attanzio et al. [74] found significantly lower TPC, TFC, and AOA in Manuka honey than in ferula (*Ferula communis*), dill (*Anethum graveolens*), and honeydew honeys from Sicily, a region of southern Italy, but comparable levels to those found in honey from eucalyptus, a species belonging to the same Myrtaceae family as Manuka. Deng et al. [17] demonstrated that Chinese buckwheat honey possesses higher phenolic content and better antioxidant capacity than Manuka honey. Marshall et al. [103] compared Manuka honey with other monofloral and multifloral honeys from Florida, finding that Manuka honeys had the highest average TPC and AOA values of all honeys, which were proportional to the darkness of the honeys. Finally, Bolanos de la Torre et al. [88] found that the TPC and antioxidant capacity of Manuka honey was directly related to the UMF rating.

Another key factor affecting honey composition is its entomological origin. Honeys produced by distinct bee species or collected from different locations possess different active compounds, and consequently exhibit differences in biological properties [81]. Several studies have compared the antioxidant properties of honey produced by stingless bees with honey produced by *A. mellifera* [81,97], or by different species of stingless bees [14,60,82,83,89,113,114]. *Melipona beecheii* honey showed the highest values for total antioxidant capacity and total phenolic, flavonoid, carotenoid, ascorbic acid, free amino acid, and protein content compared to *A. mellifera* honey [81], while *Trigona* spp. honey yielded higher total phenolic content than *Apis* spp. [97], confirming that stingless bee honey possesses higher levels of antioxidant and biological activity than *A. mellifera* honey, as reported by Avila et al. [12]

## 5. Pollen and Bee Bread

Bee pollen results from agglutination, by nectar and honeybee enzymes (e.g., amylase, catalase), of pollen grains collected from flowers by bees. Bees add small amounts of salivary secretions, nectar and/or honey to the pollen grains. Bee pollen is referred to as the ‘‘only perfectly complete food’’ as it contains all the essential amino acids needed by the human organism [118]. Bee pollen contains proteins (5–60%), reducing and non-reducing sugars (13–55%), lipids (4–7%), crude fibers (0.3–20%), essential amino acids, minerals, and bioactive substances including vitamins, enzymes, and phenolic compounds, mainly flavonoids (3–8% dry weight). Bee pollen AOA seems to be mainly related to phenolic acids, such as gallic, vanillic, protocatechuic, and *p*-coumaric acids, and flavonoids, such as quercetin, caffeic acid, caffeic acid phenethyl ester, rutin, pinocembrin, apigenin, chrysin, galangin, kaempferol, and isorhamnetin [35,119,120]. These compounds influence the grain’s visual appearance (pigmentation) and flavor (astringency and bitterness) [121]. However, the composition and antioxidant effect of bee pollen is species-specific, depending strongly on plant source together with biogeographical (regional) origin, ecological habitat, season, and entomological origin [89,118,120]. Additionally, beekeepers can introduce other changes to the chemical composition of bee pollen during cleaning, dehydration, packaging, and conservation procedures applied to fresh pollen to increase pollen shelf life [122,123]. Collection of this natural product is a relatively recent development, depending primarily on pollen being scraped off bees’ legs as they enter the hive [118]. 

The term “bee bread” refers to the pollen stored by bees in their combs, sealed with a thin layer of honey and beeswax, and matured in a beehive [124,125]. Worker bees use it as food for the larvae, and for the production of royal jelly by young bees [21]. Bees process bee bread for storage by adding various enzymes and honey, resulting in fermentation. This type of lactic acid fermentation renders the end product more digestible, as cell walls are partly destroyed during fermentation [125] and enriched with new nutrients. Its higher free amino acid content and easily assimilated sugars make it more nutritious than bee pollen [124]. One advantage of bee bread is its almost unlimited storability compared with dried or frozen pollen in which nutritional values are rapidly lost [126].

Table 3 summarizes the results of the most recent publications on pollen and bee bread AOA [6,21,89,118,121,123,124,127,128,129,130,131,132,133,134,135,136]. As indicated for honey, there is usually a positive correlation between TPC, TFC and AOA, but similar TPC and TFC content does not always correspond to similar antioxidant capacity [43,78], as the overall antioxidant capacity of each sample is the result of the combined activity of phenolic and nonphenolic compounds [21]. While honey is more frequently diluted in water for analytical purposes, pollen and bee bread are extracted using large volumes of solvent, then dried and reconstituted to the desired concentration. Ethanolic extraction was found most frequently, followed by methanolic and hydromethanolic extraction. Jin et al. [30] studied the antioxidant properties of water and methanol extract from Linden bee pollen, finding that methanol extract potentiated the antioxidant effect. Borycka et al. [136] compared the results of different extractions using water, ethanol, and methanol, analyzing five types of commercial bee pollen products: bee pellets, micronized bee pellets, pollen tablets, bee bread, and bee bread in honey. They concluded that the extraction method seemed to be crucial and that ethanol was the most effective solvent. TPC and AOA, as determined by FRAP and ABTS assays, was highest in the ethanol extracts taken from each investigated product, followed by methanol and water. Bee bread displayed the highest AOA and phenolic content compared to the other pollen products. Su et al. [132] investigated the AOA of the crude extracts but also different partitioned fractions (petroleum ether, ethyl acetate, *n*-butanol, and water fractions) of different floral sources of bee pollen. Each ethyl acetate fraction of four bee pollens had a higher AOA than the extract and other fractions. Kaškonienė et al. [125] also compared natural with “fermented” pollen to determine the different antioxidant potential of pollen and bee bread, showing that fermentation had a positive effect on antioxidant properties. The increase in biologically active compounds is assumed to be the consequence of partial destruction of the pollen cell walls by bacteria added during fermentation, as occurs naturally in the fermentation of bee bread in the hive.

Another point of issue is the treatments, such as cleaning, freezing, dehydration, and packaging, that pollen must undergo to best maintain its properties and increase its shelf life prior to sale. De-Melo et al. [119] investigated the effect of using an electric forced-air-circulation oven compared with a lyophilizer on the physical and chemical characteristics of bee pollen, measuring TPC and AOA (with DPPH and ORAC methods), among others. Both parameters were higher in lyophilized samples, which could be related to reactions occurring during heating with air circulation, such as oxidation of some compounds, for example phenols. For pollen conservation purposes, freezing is also to be preferred over dehydration by heat, as natural antioxidants are better preserved [122].

## 6. Propolis

Unlike honey and pollen, propolis (bee glue) is not a food. Bees adopt it as both a building material and a defensive substance. They use it to repair combs, reinforce the thin edges of the honeycomb, but also for its biological action, i.e., as a sealant material to prevent microorganisms (fungi and bacteria) from entering the hive, and to create the most sterile environment known in nature [137]. Moreover, it contains the putrefaction of “embalmed” intruders, killed in the hive but too large to be carried out [3]. To produce propolis, bees collect resinous materials, produced by various botanical processes in different parts of plants, and mix it with wax. It is the bee product with the highest content of specialized plant metabolites (at least 50% of its weight). It is not therefore surprising that propolis, originating from protective plant secretions and serving as a defensive substance in the beehive, is also active against human pathogens [31].

In general, propolis in nature is composed of 50% resin and vegetable balsam (including phenolic compounds), 30–40% wax and fatty acids, 5–10% essential and aromatic oils, 5% pollen, and approximately 5% other substances, including amino acids, micronutrients, and vitamins (thiamin, riboflavin, pyridoxine, vitamins C, and E) [3,138]. Different types of propolis are reported in the literature: i) poplar type (*Populus* spp., originating mainly from Europe and non-tropical regions of Asia, New Zealand, and North America); ii) birch type (*Betula verrucosa,* coming from Russia); iii) green type (*Baccharis* spp., characteristic of Brazil); iv) red type (*Dalbergia* spp., found in Brazil, Mexico and Cuba); v) Clusia type (from *Clusia* spp., from Cuba and Venezuela); vi) Pacific type (*Macaranga tanarius,* originating from Indonesia, Taiwan and Okinawa Prefecture); and vii) (the most recent) Mediterranean type (plants mainly from the Cupressaceae family found in Greece, Sicily, and Malta) [3,139]. Since these types of propolis are classified by their botanical and geographical origins and their climatic zones, their chemical composition and consequently their antioxidant content, will differ.

Table 4 summarizes the results from studies on the antioxidant capacity of propolis [6,32,55,140,141,142,143,144,145,146,147,148,149,150,151,152,153,154,155,156,157]. The extraction of bioactive compounds depends on the type and quantity of solvent, on temperature and time, and on the process used to interact with raw propolis. The results are often not univocal with respect to the extraction solvent and Table 4 presents the different solvents used and tested for extraction purposes. Among these, a mixture of ethanol and water (70/30 or 80/20) is the most commonly used. Preference should be given to this mixture as it is non-toxic and efficient in extracting polyphenols and flavonoids. Miguel et al. [150] compared water, methanol, and 70% ethanol as extraction solvents, choosing a hydroalcoholic mixture to extract phenols in propolis samples, given its good performance and lower toxicity compared to methanol. Cavalaro et al. [143] also studied the effects of ethanol/water concentration, solid-solvent ratio, and extraction time with regard to the TPC and antioxidant capacity of green Brazilian propolis, using ultrasound-assisted extraction. They optimized the procedure using 99% ethanol solution and a 1:35 propolis: solvent ratio (*w/v*), over 20 minutes. Kocot et al. [3] summarized the results of a study on the dependence between the solvent used to extract propolis and bee pollen and their antioxidant properties, reporting that despite numerous differences in composition, propolis extracts always possessed antioxidant properties, even aqueous extracts.

Several studies are underway to define the best extraction procedure: maceration is traditionally used, but in recent years sonication and microwaves have also been recommended on account of their efficiency, time saving potential and selectivity [138]. Ultrasound-assisted extraction, for example, allows many compounds to be extracted in less time (avoiding overnight steps), with fewer organic solvents, and at lower temperatures (which is key to preventing thermal degradation of some active compounds). Attention must also be paid to sonication time (since the ultrasound process can induce degradation of phenols in the sample), which is optimally set at 20–30 minutes [143,146]. The antioxidant properties of other bee products are highly dependent on many factors, such as bee species, plant origin, geographical location, temperature variation, seasonality, and storage conditions [3,141,147,153].

## 7. Beeswax

Worker bees secrete beeswax through wax glands located in the abdomen. Production of this substance generally peaks during the colony growth phase in late spring, and is used to make combs [158]. Beeswax is synthesized starting from honey sugars, and has a crystalline structure suited to hive construction. Chemical composition varies among bee species and geographical zones, and includes hydrocarbons, free fatty acids, and free fatty alcohols, linear wax monoesters, hydroxymonoesters deriving from palmitic, 15-hydroxypalmitic and oleic acids, and complex wax esters containing 15-hydroxypalmitic acid and diols [158,159]. Beeswax is used as an additive in different industrial products and processes, as in the food industry, cosmetics, and candles. In pharmaceutical preparations, it is used as a thickener, binder, drug carrier, and a release retardant [35], but several recent studies have reported the therapeutic effects of honeycombs on dental caries and toothache, and other antimicrobial properties [160]. The only studies exploring the AOA of beeswax relate to the by-products of wax recycling and the associated cost-benefit tradeoff. In bee product processing and production, honey, propolis, pollen, and royal jelly are classified as commodities, while honeycomb is discarded as a by-product. Through recycling, beeswax can be transformed into substances previously considered industrial waste, but which could be of great value in biomedicine. This has not yet been adequately explored. Zhao et al. [161] and Giampieri et al. [20,162] did, however, demonstrate that by-products from beeswax recycling are a rich source of proteins, minerals, and polyphenols, conferring strong total antioxidant capacity, and low levels of toxins. Table 5 summarizes the results of their research on the antioxidant properties of beeswax derivatives. Both research teams affirmed that the antioxidant capacity of wax extracts is higher than that of (some) honey. In Table 5 also royal jelly and bee venom antioxidant properties are considered [20,29,161,162,163,164,165,166].

## 8. Royal Jelly

Royal jelly is a substance secreted by the hypopharyngeal and mandibular glands of worker honeybees. It is a yellowish, creamy, acidic substance with a slightly pungent odor and taste composed, on a wet weight basis, of water (60–70%), proteins (9–18%), sugars (7–18%)-mainly fructose, glucose and sucrose-lipids (3–8%), minerals (0.8–3.0%), ash (0.8–3%), and traces of polyphenols and vitamins [163]. Royal jelly is fed to all bee larvae in the early stages of life and to the queen bee until she dies. It plays a crucial role in determining the caste of honeybees because larvae fed with greater amounts of royal jelly for a longer period develop into large, fertile, long-living queens rather than smaller, infertile, short-living workers [167]. Royal jelly has been proven to have numerous functional properties such as disinfectant action, and antibacterial, anti-inflammatory, vasodilative, hypotensive, antihypercholesterolemic, antitumor, and antioxidant activity, as reported in the review by Ramadan and Al-Ghamdi [168]. The antioxidant potency of royal jelly is attributed to its polyphenolic and flavonoid compounds; free amino acids, including essential ones; small peptides, such as di-peptides (Lys-Tyr, Arg-Tyr, and Tyr-Tyr) obtained from protease hydrolyzed royal jelly proteins; peptides and proteins; fatty acids (the main being 10-hydroxydecanoic acid); and vitamins [3,162,169]. The principal flavonoids present in royal jelly include flavonoles (e.g., quercetin, kaempherol, galangin, and fisetin), flavanones (e.g., pinocembrin, naringin, and hesperidin), and flavones (e.g., apigenin, acacetin, chrysin, and luteolin) [168]. Major royal jelly proteins (MRJPs) represent 83–90% of the protein component of royal jelly and are composed of nine known members with molecular weights of between 49 and 87 kDa [28,167]. Table 5 summarizes the results of the most recent studies on the antioxidant properties of royal jelly and its derivatives. Using a DPPH radical-scavenging assay, Park et al. [29] confirmed the antioxidant capacity of MRJPs 1–7 of *A. mellifera* at levels ranging between approximately 30 and 80% of residual radical. Pavel et al. [162] quantified TPC and AOA in local (Romanian) and commercial royal jelly (10% in distilled water). TPC was measured by a Folin-Ciocalteu reagent reduction and found to be 23.49 and 23.25 mg GAE/g for local and commercial royal jelly, respectively. Antioxidant capacity was determined using DPPH radical scavenging and FRAP assays, resulting in 37.23 and 35.94% inhibition and 2.20 and 1.83 mM Fe^2+^/g, respectively. Hence, no major differences were found between the two types of royal jelly. There is a paucity of data in the literature on the polyphenolic content and AOA of royal jelly, and existing data are not very recent. TPC values ranged from 21.2 to 22.8 mg/g of powder lyophilized from water and alkaline extracts [170], and from 150 to 219 µg/g for different royal jelly samples [171]. However, the data are not comparable as different extracts and different formulations of royal jelly were used (fresh or lyophilized). While the polyphenolic content and AOA of royal jelly do appear to be highly variable, new targeted studies are nonetheless warranted. Conversely, several in vivo studies in animal models and humans have been performed showing the antioxidant potential of this functional food, as reviewed in Kunugi et al. [169] and Sığ et al. [172].

## 9. Bee Venom

Bee venom, or apitoxin, is a mixture of several components with proven therapeutic benefits. The main components are peptides, such as melittin (which is also the main component of bee venom) and apamin, and proteins (enzymes), followed by low molecular compounds, including phospholipids, biogenic amines (such as histamine and catecholamines), amino acids, sugars, volatiles (pheromones), and minerals [35,173]. Bees use their venom as a defense tool against predators, intruders, and for colony defense, but the healing properties of bee stings have been known since ancient times [174]. Evidence has recently been found to support these medical claims in numerous studies and the use of bee venom is applied in different conditions with various patho-physiological substrates, including for the nervous, immune, or cardiovascular systems [163].

As shown in Table 5, only three recent studies have quantified the AOA of bee venom using classical assays. All samples revealed antioxidant properties, which were apparently unrelated to any of the individual components identified and quantified in the same samples. Some data suggest that melittin alone exerts very poor AOA compared to bee venom extracts and this might be due to the influence of other venom components [163]. Hence, some other minor compounds, together with synergistic/antagonistic effects at specific concentrations, could be involved in the reported bioactivities, contributing to different results among bee venom samples [164,165].

As in the case of royal jelly, several publications can be found on studies of AOA of bee venom in vivo in animal models and humans [174,175,176,177,178,179], and in vitro in cell cultures, as shown in Table 6.

## 10. In Vitro Determination of AOA

Different studies have been carried out to determine in vitro the AOA of bee products using biological (cellular) systems. The more recent ones are summarized in Table 6 [16,17,18,19,20,21,22,23,24,25,26,27,28,29,30,32,33,34,47,74,118,142,161,165,180]. Cell lines of different types and origins have, in the main, been used for this purpose in all bee products. Propolis was the most widely tested. The various systems adopted performed well in determining the AOA of bee products and different analytical methods from the ones reported in Table 1 were applied. In addition, other effects of bee products on cell lines and in in vitro systems have also been tested in parallel but are beyond the scope of this review.

## 11. Concluding Remarks

The antioxidant properties of different bee products can be only be compared when the data are obtained using the same methods and units of measurement for the different matrices. Bartkiene et al. [6] compared honey, propolis, and bee bread, ranking TPC and AOA values (measured as % DPPH) in the following order: bee bread > propolis > honey, and bee bread > honey > propolis, respectively. Based on data from the literature, propolis should be the most powerful antioxidant of bee products—having been shown to contain the highest levels of phenols and flavonoids—followed by pollen and royal jelly [3,35]. The results do, however, differ considerably depending on matrix, extraction solvent, and assay. By way of example, Nakajima et al. [22] (using an antioxidant-capacity assay to measure the radicals induced in a rat cell line through application of ROS) observed the rank order of antioxidant effects to be as follows: propolis water extract > propolis ethanol extract > pollen, but neither royal jelly nor 10-hydroxy-2-decenoic acid (10-HDA) had any effect. A comparative study of the AOA of honey and propolis performed by Mouhoubi-Tafinine et al. [55] showed propolis samples to have higher concentrations of polyphenols, flavonoids, vitamin C, and carotenoids, and to display a greater AOA (measured by the reducing power assay). Even compared to pollen, honey clearly appears to have lower phenol and AOA levels, as shown by Duarte et al. [89] Mohdaly et al. [181] reported that propolis extract had superior scavenging activity (based on DPPH and ABTS assays) compared to pollen extract. The disparity of the results presented in this review is well known to be influenced by considerable botanical, geographical, and other above-mentioned differences among samples. There are many inconsistencies in the information related to AOA analysis of bee products, such as sample dilution, extraction method, and conditions, quantification method, and criteria for reporting the results. All these have a decisive influence on the disparity of results, hindering comparison of the biological properties of different samples of the same bee products, despite being similar. Hence, to determine valid common criteria, the analytical procedures need to be as standardized as possible to accurately classify bee products by composition and commercial value. It is difficult to compare the analytical results reported in this review with each other, because even where the same analytical technique is used (as the Folin-Ciocalteu method), the results may be expressed in different units. Results are calculated by plotting the concentration of a calibration standard against absorbance on a standard curve, but analytical results can only be compared with others when the same reference compounds are used. Furthermore, bee products are chemically very complex, and the use of solvents of different polarity affects the composition of the solutions or extracts to be analyzed. While hydrophilic substances are more soluble in polar solvents such as alcohols, hydrophobic ones show greater affinity for non-polar solvents such as hydrocarbons. The analytical result can therefore also vary according to the solvent used to dissolve the honey or propolis or other hive products being tested. The extract’s properties strongly depend on the solvent used but also on extraction conditions, time, and temperature [3]. Accurate standardization of analytical methods is needed to define quality criteria and support estimation of the commercial value of these expensive natural products. Working with standardized methodologies, accepted by researchers and analytical laboratories, with adequate analytical protocols that define the solvents, extraction procedures, and criteria for expressing the results, will allow the collection of reliable, comparable data.

## Figures and Tables

**Table 1:** Summary of the assays used for antioxidant capacity determination.

Assay	Reaction Mechanisms	Methods in Brief	Main Characteristics	Set up Method Reference
Total phenolic (phenols and polyphenols) content (TPC)	Electron transfer	Reduction of a yellow molybdate-tungstate reagent (Folin-Ciocalteu reagent) induced by the phenols in the sample, under alkaline conditions, a blue-colored chromophore (abs. 700, 740, 750, 760 or 765 nm).	Simple, rapid, and reproducible method. Sensitive to nonphenolic electron donating antioxidants as reducing sugars, amino acids, ascorbic acid, Cu (I) [42,43].	[44]
Total flavonoids content (TFC)	Colored complex formation	Aluminum chloride forms acid stable yellow complexes with the C-4 keto groups and either the C-3 or the C-5 hydroxyl group of flavones and flavonols. In addition, it forms acid labile complexes with the orthodihydroxyl groups in some flavonoid rings (abs 420 or 510 nm).	Possible overestimation as some nonflavonoid compounds exhibit absorbance at the same wavelength. Specific only for flavones and flavonols [45].	[46]
DPPH (1,1-diphenyl-2-picrylhydrazyl) free radical-scavenging assay	Electron transfer	The decolorization of DPPH occurs from purple to yellow when the unpaired electron of DPPH forms a pair with a hydrogen donated by a free radical scavenging antioxidant, thus converting DPPH into its reduced form (abs. 515 or 517 nm).	Easy, simple, rapid, reproducible, and reasonably costly method. Efficient for thermally unstable compounds and highly sensitive [42,47]. Unaffected by metal ion chelation and enzyme inhibition [48]; reflects only the activity of water-soluble antioxidants [49]. Sensitive to light, oxygen, and impurities. Rate-limited by a proton transfer step, affected by the solvent system and the ionization equilibrium of phenol and phenolate compounds in solution [50].	[51]
Ferric reducing antioxidant power (FRAP) assay	Electron transfer	Reduction of a ferric 2,4,6-tripyridyl-s-triazine complex (Fe^3+^-TPTZ) to its ferrous, violet-blue form (Fe^2+^-TPTZ) in the presence of antioxidants (abs. 593 or 700 nm).	Simple, reproducible, and sensitive. The high amount of reducing sugars in honey could contribute to higher reducing antioxidant power. Unable to detect slowly-reacting polyphenolic compounds and thiols [48].	[52]
Cupric ion reducing antioxidant capacity (CUPRAC) Assay	Electron transfer	Bis(neocuproine)copper(II) chloride [Cu(II)-Nc], reacts with polyphenols where the reactive Ar-OH groups of polyphenols are oxidized to the corresponding quinones and Cu (II)-Nc is reduced to the highly colored Cu (I)-Nc (abs 450 nm).	Carried out at pH 7.0 and simultaneously measure hydrophilic and lipophilic antioxidants. Fast enough to oxidize glutathione and thiol-type antioxidants [53].	[54]
Reducing power method (RP)	Electron transfer/ Hydrogen atom transfer reaction.	Substances, which have reduction potential, react with potassium ferricyanide (Fe^3+^) to form potassium ferrocyanide (Fe^2+^), which then reacts with ferric chloride to form ferric ferrous complex (abs. 700 nm).	Chelating effect of the ions Fe^3+^ of polyphenols related to the highly nucleophilic aromatic rings. The degree of hydroxylation and methylation of the phenolic compound and the presence of other non-phenolic compounds such as enzymes and non-enzyme materials possibly involved [55].	[56]
Total antioxidant capacity (TAA)/ phosphomolybdenum method	Electron transfer	Based on the reduction of Mo(VI) to Mo(V) by the reducing compounds and the formation of a green phosphate/Mo(V) complex at acidic pH (abs. 695 nm).	Simple, sensitive, and cheap method to evaluate water-soluble and fat-soluble antioxidants. Bad correlation with bioactive compounds (phenolics, flavonoids) and weak correlation with free radical scavenging assays (DPPH). Non-specific, detecting also ascorbic acid, carotenoids, and α-tocopherol [42].	[57]
Ferrous ion-chelating activity	Metal-chelating activity	Ferrozine can form a complex with a red color by forming chelates with Fe^2+^. This reaction is restricted in the presence of other chelating agents and results in a decrease of the red color of the ferrozine-Fe^2+^ complexes. EDTA or citric acid can be used as a positive control (abs. 562 nm).	Bivalent transition metal ions can lead to the formation of hydroxyl radicals and hydroperoxide decomposition reactions. Iron chelation can delay these processes [58]. Simple, reproducible, and cheap but non-specific reacting also with peptides and sulphates [42].	[59]
Oxygen radical absorbance capacity (ORAC) method	Hydrogen atom transfer reaction	Measuring the decrease in fluorescence of a protein (fluorescein) that results from the loss of its conformation when it suffers oxidative damage caused by a source of peroxyl radicals (ROO^•^) generated by the thermolytic breakdown of 2,2′-azobis(amidinopropane) dihydrochloride (AAPH) (excit. 485 ± 20 nm emiss. 528 ± 20 nm).	Both hydrophilic and hydrophobic antioxidants detected by altering the radical source and solvent. Use reactants with a redox potential and mechanism of reaction similar to those of physiological oxidants at a physiological pH. The most biologically relevant assays [60].	[61]
ABTS (2,2-azino-bis(3-ethylbenzthiazoline-6-sulfonic acid)) radical cation decolorization assay/Trolox equivalent antioxidant capacity (TEAC) method	Electron transfer	When an antioxidant is added to the ABTS^•+^ blue-green chromophore, it is reduced to ABTS and discolored (abs. 734 or 750 nm).	A “nonphysiological” radical source used over a wide pH range and in multiple media to determine both hydrophilic and lipophilic antioxidant capacities [60].	[62]
Superoxide radical (SOD) scavenging activity assay	Superoxide scavenging potential	Superoxide radicals are produced by NADH/PMS (phenazine methosulfate) systems via the oxidation of NADH, bringing about the reduction of nitroblue tetrazolium (NBT) to purple formazan (abs. 560 nm).	Dangerous hydroxyl radicals and singlet oxygen are produced by superoxide anions, both contributing to oxidative stress [63]. They bear resemblance to biological systems in contrast to DPPH or ABTS, which are synthetic radicals. Non-specific and expensive [42].	[64]
Hydroxyl radical scavenging activity assay	Hydroxyl radical scavenging potential	Based on the competitive ability of the sample with deoxyribose for hydroxyl radicals generated from Fe^3+^-EDTA-ascorbic acid and H_2_O_2_ reaction mixture, leading to a decreased yield of malondialdehyde-like products, which in turn reduce the formation of the TBA-chromophore (abs. 520 or 532 nm).	Hydroxyl radical, one of the potent reactive oxygen species, reacts with polyunsaturated fatty acid moieties of cell membrane phospholipids, damaging the cell [63].	[65]
Hydrogen peroxide scavenging activity assay	Hydrogen peroxide scavenging potential	The absorbance of a solution of hydrogen peroxide in phosphate buffer (PBS) is acquired before and after the addition of the sample (abs. 230 nm).	Hydrogen peroxide may enter into the human body through inhalation and eye or skin contact. Rapidly decomposed into oxygen and water; may produce hydroxyl radicals that can cause DNA damage [63].	[66]
β-Carotene-linoleic acid bleaching assay (BCB)	Hydrogen transfer reaction	Linoleic acid is oxidized by ROS produced by oxygenated water. The products will initiate the β-carotene oxidation, and, as the molecule loses its double bonds, the compound loses its characteristic orange color (abs. 434 nm).	Hydrogen transfer reactions are solvent and pH-independent and usually quite rapid (seconds to minutes). Reducing agents, including metals, complicate these assays leading to erroneously high reactivity [67].	[68]

**Table 2:** Summary of the results from studies on antioxidant capacity in honey.

Sample Size	Botanical Origin	Bee Species ^1^	Country	TPC ^2^	TFC ^3^	AOA ^4^	Characterization	References
26	10 Sumer, 10 Sidr and 6 multiflora	*A. mellifera*	Oman	842–2898 mg GAE/kg	521–2890 mg CE/kg	7.8–190.1 mg/mL EC50 DPPH	n.d.	[78]
11	1 Talh, 1 Olive, 1 Sidr, 8 multiflora	n.s.	Saudi Arabia	0.78–5.02 mg GAE/g	n.d.	5.89–53.93% DPPH	GC-MS	[79]
83	17 Linen vine, 16 Morning glory, 18 Christmas vine, 16 Black mangrove, 16 Singing bean	n.s.	Cuba	213.9–595.8 mg GAE/kg	10–25 mg CE/kg	27.0–96.9 µmol TE/100g FRAP 103.5294.5 µmol TE/100g TEAC	n.d.	[80]
16	Multiflora, 8 from *A. mellifera* and 8 from *M. beecheii*	*A. mellifera*, *M. beecheii*	Cuba	54.30 and 94.39 mg GAE/100g	2.68 and 4.19 mg CE/100g	159.70 and 175.82 µmol TE/100g FRAP 31.06 and 42.23 µmol TE/100g DPPH	HPLC-DAD-ESI-MS/MS	[81]
32	Different monoflora and 2 honeydew	*A. mellifera* spp. *sicula*	Italy	16.5–133.3 mg GAE/100g	4.0–82.1 mg QE/100g	17.8–165.7 mg AAE/100g FRAP 8.5–238.4 μmol TE/100g DPPH 19.2–270.3 4 μmol TE/100g ABTS	n.d.	[74]
32	Multiflora, 8 for each bee species	*M. bicolor*, *M. quadrifasciata*, *M. marginata*, *S. bipuncata*	Brazil	220.4–708.1 mg GAE/kg	n.d.	1.61–34.73 µmol TE/kg ABTS 9.71–39.10 µmol TE/kg DPPH 35.49–94.35 µmol TE/kg ORAC	HPLC–PDA	[60]
14	n.s.	n.s.	Lithuania	168–278 mg GAE/100g	n.d.	65–88% DPPH	n.d.	[6]
8	Multiflora from 6 different Meliponinae	Meliponinae, 6 spp.	Brazil	10.4–57.4 mg GAE/100g	n.d.	0.8–28.2 mg AAE/100g DPPH 67.5–734.5 µmol Fe^2+^/100g FRAP	LC-MS	[82]
33	Multiflora from 10 different Meliponinae	Meliponinae, 10 spp.	Brazil	10.3–98.0 mg GAE/100g	n.d.	1.41–18.5 mg AAE/100g DPPH 61.1–624 µmol Fe^2+^/100g FRAP	n.d.	[83]
13	Multiflora from 9 different Meliponinae	Meliponinae, 9 spp.	Brazil		n.d.	199–667 μmol TE/100g ORAC	HPLC–ESI-MS/MS	[14]
14	8 Rape and 8 multiflora	n.s.	Hungary	170–330 mg GAE/kg	n.d.	63–110 μmol TE/100g TEAC 27–42 mg TE/mL EC50 DPPH 22–39 μmolTE/g ORAC	n.d.	[84]
20	Avocado	n.s.	Spain	103.1–137.8 mg GAE/100g	n.d.	2.4–2.8 μmol TE/g TEAC	n.d.	[85]
62	11 monoflora, 2 honeydew and 7 multiflora	n.s.	Turkey	16.02–120.04 mg GAE/100g	0.65–8.10 mg QE/100g	0.64–4.30 μmol Fe^2+^/g FRAP 12.56–152.40 mg TE/mL EC50 DPPH	HPLC-UV	[36]
16	16 monoflora	n.s.	China	60.5–100.8 mg GAE/100g	0.6–2.3 mg RE/100g	56.0–101.2 mg TE/100g DPPH 10.1–14.5 mg TE/100g ABTS 7.0–14.9 mg TE/100g FRAP	n.d.	[86]
15	8 monoflora, 7 multiflora	n.s.	Spain	23.1–158 mg GAE/100g	1.65–5.93 mg CE/100g	5.46–202 mg/mL EC50 DPPH 26.3–215 mg/mL EC50 RP −1.34–92.9 % BCB	HPLC-UV	[43]
7	Multiflora	*M.(Michmelia) seminigra merrilae*	Brazil	17.0–66.0 mg GAE/g	n.d.	210–337 mg TE/mL EC50 ABTS	HPLC-DAD	[87]
4	Manuka	n.s.	New Zealand	372–576 mg GAE/kg	n.d.	545–756 μmol Fe^2+^/100g FRAP	n.d.	[88]
460	Monoflora	n.s.	Italy	107.2–564.2 mg GAE/kg	33.1–213 mg QE/kg	3.4–161.3 mg/mL EC50 DPPH 24.4–72.8 μM AAE/g FRAP	LC-MS	[9]
31	Multiflora	Meliponinae, 7 spp.	Brazil	32–136 mg GAE/g	8–55 mg QE/g	DPPH, BCB, FRAP (graphicated)	n.d.	[89]
20	Buckwheat	n.s.	Poland	181–355 mg GAE/100g	8.0–30.4 mg QE/100g	51–95.2% DPPH 195–680 μmol TE/100g FRAP	UPLC-PDA-MS/MS	[90]
90	44 monoflora, 29 honeydew and 17 multiflora	n.s.	Poland	254.5–1353.7 mg GAE/kg	n.d.	21.81–82.41% DPPH 656.73–3635.49 µmol TE/kg FRAP	n.d.	[48]
8	Carob	n.s.	Morocco	75.5–245.2 mg GAE/100g	2.26–4.79 mg QE/100g	35.03–60.94 mg AAE/g TAA 12.54–23.52 mg/mL EC50 DPPH 1.9–4.4 mg AAE/mL EC50 FRAP	n.d.	[91]
187	34 chestnut, 17 eucalyptus, 31 blackberry, 10 heather, 13 honeydew and 82 multiflora	n.s.	Spain	78.4–181 mg GAE/100g	4.3–9.6 mg QE/100g	9.5–17.8 mg AAE/mL EC50 DPPH	n.d.	[92]
32	Honeydew	n.s.	Spain	79.5–187 mg GAE/100g	6.6–13.1 mg QE/100g	52.9–95.6% DDPH	n.d.	[93]
7	Forest, pine, urtica, meadow, linden, 2 acacia	n.s.	Serbia, Germany, Greece	94.0–620.7 µg GAE/ml	n.d.	0.2–4.98 µmol TE/g FRAP 5.9–12.9 µmol TE/g ORAC 1.0–5.82 µmol TE/g ABTS 0–1.21 µmol TE/g EC50 DPPH	n.d.	[94]
23	Monoflora	n.s.	Turkey	45.4–470.7 mg GAE/100g	n.d.	12.01–65.52 mg/mL EC50 DPPH 0.0022–0.0091 mg TE/100g FRAP 32.09–94.87% BCB.	n.d.	[95]
22	20 monoflora, 2 honeydew	n.s.	Poland	3.43–22.33 mg GAE/100g	n.d.	41.42–83.16 mg GAE/100g ABTS	HPLC-DAD	[77]
40	Honeydew “dryomelo”	*A. mellifera*	Greece	1221–1495 mg GAE/kg	n.d.	56.8–72.4% DPPH	n.d.	[96]
11	2 tualang, 2 gelam, 2 pineapple, 2 borneo (*Apis* spp.) and 3 kelulut (*Trigona* spp.)	*Apis* spp. and *Trigona* spp.	Malaysia	590.5 and 784.3 mg GAE/kg	n.d.	n.d.	n.d.	[97]
4	Tualang, gelam, indian forest, pineapple	n.s.	Malaysia	27.75–83.96 mg GAE/100g	24.74–50.45 mg QE/100g	16.12–53.06 mg AAE/g TAA 5.80–10.86 mg/mL EC50 DPPH 47.92–121.89 μmol Fe^2+^/100g FRAP	n.d.	[98]
28	4 black locust, 5 buckwheat, 4 lime, 2 goldenrod, 3 heather, 10 rapeseed	n.s.	Poland	121.6–1173.8 mg GAE/kg	n.d.	0.6–6.7 FRAP mmol Fe^2+^/kg 0.2–1.4 mmol TE/kg DPPH	HPLC-DAD	[99]
40	Multiflora, lime, rape, raspberry, mixture, honeydew	n.s.	Czech	82.5–242.5 mg GAE/kg	n.d.	141.52–407.08 mg AAE/kg DPPH 489.44–982.93 mg AAE/kg ABTS 295.35–776.05 mg AAE/kg FRAP	n.d.	[100]
9	5 orange and 4 multifloral	n.s.	Brazil	40.36 and 58.05 mg GAE/100g	0.17 and 1.53 mg QE/100g	38.54 and 16.62-mg/mL EC50 DPPH	HPLC-DAD	[101]
6	B. pilosa, D. longan, L. chinensis, C. maxima, A. formosana, and 1 multiflora	n.s.	China	0.31–0.82 mg GAE/g	29.7–124 mg QE	15.2–84.9% DPPH	n.d.	[102]
20	Monoflora, multiflora and Manuka	n.s.	Florida, New Zealand	286–1080 µg GAE/g	n.d.	0.28–2.1 µmol TE/g DPPH 1.48–18.2 µmol TE/g ORAC	HPLC-UV	[103]
4	n.s.	n.s.	Algeria	15.84–61.63 mg GAE/100g	2.07–10.15 mg CE/100g	RP (graphicated)	n.d.	[55]
20	4 multiflora, 4 linden, 4 rapeseed, 2 sunflower, 1 phacelia, 3 acacia and 2 honeydew	n.s.	Serbia	n.d.	n.d.	22.96–79.45% DPPH	n.d.	[72]
49	28 eucalyptus, 6 Japanese grape, 5 mastic, 3 quitoco, 1 wildflower, 6 multiflora	*A. mellifera*	Brazil	26.0–100.0 mg GAE/100g	0.65–8.10 mg QE/100g	1.28–18.48 µmol TE/g ORAC 25.45–294.26 mg/mL EC50 DPPH 0.22–2.11 µmol TE/g FRAP	HPLC-UV	[104]
37	11 apple, 8 cherry, 8 saffron and 10 wild bush	n.s.	India	37–117 mg GAE/100g	8–17 mg QE/100g	55–84% DPPH 19–51 mg AAE/100g DPPH	HPLC-DAD	[105]
24	7 acacia, 8 pine, 9 multiflora	n.s.	India	22.68–59.84 mg GAE/100g	6.10–8.12 mg QE/100g	52.27–55.37% DPPH 14.13–23.74 mg AAE/100g DPPH	n.d.	[106]
16	Monoflora	n.s.	Turkey	170.06–885.43 mg GAE/100g	n.d.	0.27–2.56 mg/mL EC50 DPPH 0.51–0.62 mmol TE/g	n.d.	[107]
45	4 thyme, 10 rape, 10 mint, 6 raspberry, 9 sunflower, 6 multiflora	n.s.	Romania	18.91–23.71 mg GAE/100g	17.45–33.58 mg QE/100g	55.49–79.05% DPPH	HPLC-DAD	[75]
78	16 chestnut, 14 eucalyptus, 12 citrus, 18 sulla and 18 multiflora	n.s.	Italy	10.82–14.67 mg GAE/100g	5.09–14.05 mg QE/100g	58.40–60.42% ABTS 152.65–881.34 µM Fe^2^ FRAP 54.29–78.73% DPPH	n.d.	[39]
14	Monoflora and 5 multiflora	n.s.	Mexico	283.9–1142.9 mg GAE/kg	n.d.	910.2–2927.4 µmol TE/kg ABTS 81.9–255 µmol TE/kg DPPH 749.4–3097.1 µmol Fe^2+^/kg FRAP	n.d.	[108]
91	53 chestnut and 38 honeydew	n.s.	Spain	125 and 128 mg GAE/100g	8.4and 9.4 mg QE/100g	58.4–68.4% DPPH	n.d.	[109]
129	Loco, opoponax-tree, alfalfa, barberry, thyme, argentine thistle and dill	n.s.	Iran	33.34–259.52 mg GAE/kg	n.d.	204.14–1383.18 μmol Fe^2+^/100g FRAP	n.d.	[110]
39	Acacia, jujube, vitex, linden, fennel, buckwheat, Manuka	n.s.	China (mainly)	9.15–294 mg GAE/100g	6.85–64.8 mg QE/100g	n.d.	UPLC-MS/MS	[19]
50	Rhododendron	n.s.	Turkey	20.29–109.19 mg GAE/100g	n.d.	21.9–58.21 mg AAE/g TAA 36.1–90.73% DPPH	n.d.	[111]
9	Mimosoideae	*M. subnitida*	Brazil	1.2–1.3 mg GAE/g	n.d.	10.6–12.9 mg/mL EC50 DPPH 6.1–9.7 mg/mL EC50 ABTS 51.5–74.6% BCB	HPLC-DAD	[112]
11	7 from *A. mellifera* and 4 from *M. q. anthidioides*	*A. mellifera* and *M. q. anthidioides*	Brazil	47.67–341.51 mg GAE/kg	8.88- 216.29 mg QE/kg	86.76–180.28 μmol TE/L DPPH 98.43–365.35 μmol Fe^2+^/L FRAP 1.91–19.71 μmol EBHA/L BCB	n.d.	[113]
24	*Ziziphus joazeiro*, *Mimosa quadrivalvis* L., *Mimosa arenosa*, *Croton heliotropiifolius*	*M. subnitida* and *M. scutellaris*	Brazil	31.5–126.6 mg GAE/100g	1.9–4.2 mg QE/100g	11.2–46.9% DPPH 23.2–46.9 μmol TE/100g ABTS 8.9–54.3 μmol TE/100g ORAC	HPLC-DAD	[114]
20	n.s.	n.s.	Turkey	35.3–1961.5 mg GAE/100g	5.38–26.75 mg QE/100g	54.11–68.94% DPPH 58.93–110.54 mg AAE/g TAA	n.d.	[115]
64	Honeydew	n.s.	Croatia	0.57–1.6 mg GAE/g	n.d.	12.2–48.89% DPPH	UHPLC-LTQ OrbiTrap MS and HPLC-DAD-MS/MS	[116]
82	Monoflora and multiflora	n.s.	Poland	40.5–177 mg GAE/100g	n.d.	47.2–83.4% DPPH 0.64–1.46 μmol TE/kg DPPH 6–79% ABTS	n.d.	[117]

TPC: total phenolics compounds; TFC: total flavonoids compounds; AOA: antioxidant activity; n.s.: not specified; n.d.: not determined; ^1^ A.: Apis; M.: Melipona; S.: Scaptotrigona; ^2^ GAE: gallic acid equivalents; ^3^ CE: catechin equivalents, QE: quercetin equivalents, RE: rutin equivalents; ^4^ TE: Trolox equivalents, AAE: ascorbic acid equivalents, Fe^2+^: FeSO_4_*7H_2_0 equivalents.

**Table 3:** Summary of the results from studies on antioxidant capacity in pollen and bee bread.

Sample Size	Botanical Origin ^1^	Sample Type	Country	TPC ^2^	TFC ^3^	AOA ^4^	Extraction	Characterization	References
3	*Cistus creticus* L. (rock rose)	Pollen	Greece	15.2–60.2 mg GAE/g	6.0–57.6 mg QE/g	0.7–233.3% 200 µg/ml EC50 DPPH18.4–77.9% 100 µg/ml EC50 ABTS	Cyclohexane, dichloromethane, butanol and water	n.d.	[127]
1	n.s. (hives of *A. mellifera* L. bees)	Pollen	Brazil	19.69 mg GAE/g	6.81 mg QE/g	0.94 mg/ml DPPH 120.1 µmol TE/g ABTS 60.64 mmol Fe^2+^/g FRAP 91.93% BCB	Ethanol	LC-DAD	[128]
56	n.s. (hives of *A. mellifera* L. bees), palynological evaluation performed	Pollen	Brazil	6.5–29.2 mg GAE/g	0.3–17.5 mg QE/g	9.4–155 µmol TE/g DPPH 133–563 µmol TE/g ORAC	Ethanol	HPLC-PDA	[123]
25	n.s. (hives of Meliponini, 7 spp.)	Pollen	Brazil	6.9–21 mg GAE/g	0.3–17 mg QE/g	DPPH, BCB and FRAP (graphicated)	Ethanol	n.d.	[89]
3	n.s. (hives of *T. apicalis, T. itama* and *T. thoracica*)	Pollen	Malaysia	33.46–135.93 mg GAE/g	15.28–31.80 mg QE/g	0.86–3.24 EC50 mg/ml DPPH	Ethanol	n.d.	[129]
1	n.s., palynological evaluation performed	Pollen	Greece	10.49 mg PAE/g	n.d.	181.4 µg/ml EC50 DPPH	methanol	GC-MS	[130]
10	Heterofloral	Pollen	Turkey	509–1746 mg GAE/100g	n.d.	12.3–33.84% DPPH	Water	n.d.	[131]
4	Camellia, rape, rose and lotus	Pollen	China	6.82–62.35 mg GAE/g	n.d.	DPPH, RP and ABTS (graphicated)	Petroleum ether, ethyl acetate, n-butanol and water	HPLC-ESI-Q-TOF-MS/MS	[132]
5	Heterofloral, palynological evaluation performed	Pollen	Portugal	10.5–16.8 mg GAE/g	n.d.	2.16–5.87 mg/ml EC50 DPPH 3.11–6.52 mg/ml BCB	Methanol	n.d.	[133]
8	Heterofloral	Pollen	Portugal-Spain	5.57–15 mg GAE /g	n.d.	119–276.8 µM TE/g ABTS	Methanol	n.d.	[118]
13	n.s.	Pollen	Turkey	44.07–124.1 mg GAE/g	n.d.	11.77–105.06 µmol TE/g EC50 FRAP 0.65–8.2 mg/ml EC50 DPPH 33.1–86.8 µmol TE/g CUPRAC	Methanol	HPLC-UV	[134]
40	Heterofloral	Pollen	Poland	5.57–15.0 mg GAE/g	n.d.	119–276.8 µM TE/g ABTS	Methanol-water (70%, *v/v*)	Raman and FTIR	[135]
4	n.s.	Bee bread	Lithuania	306–394 mg GAE/100g	n.d.	85–93% DPPH	Ethanol	HPLC-UV	[6]
1	n.s.	Bee bread	Morocco	n.d.	n.d.	143 mg AAE/g TAA 0.19 mg/ml EC50 RP 0.5 mg/ml EC50 ABTS 0.98 mg/ml EC50 DPPH	Methanol-water (80:20 v/v)	LC-DAD–ESI/MS	[124]
5	n.s.	Bee bread	Ukraine	12.36–25.44 mg GAE/g	13.56–18.24 µg QE/g	DPPH and TAA (graphicated)	Ethanol	n.d.	[136]
3	n.s.	Bee bread	Poland	32.78–37.15 mg GAE/g	n.d.	0.56–1.1 mmol/L ABTS (Randox test)	Ethanol	GC-MS	[21]
15	n.s. (hives of *A. mellifera L*. bees)	Bee bread	Colombia	2.5–13.7 mg GAE/g	1.9–4.5 mg QE/g	35.0–70.1 mmol TE/g FRAP 46.1–76.3 µmol TE/g ABTS	Ethanol	n.d.	[121]

TPC: total phenolics compounds; TFC: total flavonoids compounds; AOA: antioxidant activity; n.s.: not specified; n.d.: not determined; ^1^ A.: Apis, T. Trigona; ^2^ GAE: gallic acid equivalents; PAE: protocatechuic acid equivalents; ^3^ QE: quercetin equivalents; ^4^ AAE: ascorbic acid equivalents; TE: Trolox equivalents; Fe^2+^: FeSO_4_*7H_2_0 equivalents.

**Table 4:** Summary of the results from studies on antioxidant capacity in propolis.

Sample Size	Botanical Origin/Bee Species ^1^	Propolis Type	Country	TPC ^2^	TFC ^3^	AOA ^4^	Extraction	Characterization	References
1	*H. itama*	n.s.	Brunei	n.d.	n.d.	12.75–317.65 mg AAE/g DPPH	Ethanol-water mixtures with different volume fractions (from 0.0 to 1.0) of ethanol (96%).	n.d.	[140]
4	n.s.	n.s.	Lithuania	211–298 mg GAE/100g	n.d.	32–80% DPPH	Ethanol	HPLC-UV	[6]
2	n.s.	green and brown	Brazil	31.88–204.30 mg GAE/g	n.d.	21.50–78.77 µg/mL EC50 DPPH	Ethanol- hexane-dichloromethane	GC-MS	[141]
1	*M. orbignyi*	n.s.	Brazil	211 mg GAE/100g	23 mg QE/100g	40 µg/mL EC50 DPPH	Ethanol (80%)	n.d.	[32]
6	*A. mellifera*	n.s.	Chile	1.3–1.6 µM CAE/mg	n.d.	0–7.3 µM CAE/mg ORAC-PGR 8.9–33.1 µM CAE/mg ORAC-FL 1.8–3.2 µM CAE/mg FRAP	Ethanol (90%) “wax free”	HPLC-UV-ESI-MS/MS	[142]
1	n.s.	green	Brazil	57.9–1614.8 mg GAE/g	n.d.	21.3–13244.5 µmolTE/g ORAC 408.6–13412.1 µmol TE/g ABTS	Best using 99% ethanol solution, 1:35 propolis:solvent ratio (w/v), over 20 min	n.d.	[143]
33	n.s.	n.s.	Brazil	n.d.	n.d.	61.9–1770 µmol Fe^2+^/g FRAP	Ethanol (80%)	FTNIR	[144]
1	n.s.	n.s.	Brazil	n.d.	n.d.	14.95–112.12 mg QE/g DPPH 0–36.28 mg QE/g β-carot	Hexane, chloroform, ethyl acetate and methanol	GC–EI-MS HPLC–DAD–ESI-MS/MS and NMR	[145]
6	*A. mellifera*	n.s.	3 Romania, 2 Spain, 1 Honduras	97–442 mg GAE/g	n.d.	n.d.	Ethanol (70%)	HPLC-UV	[146]
10	*M. mondury, M. quadrifasciata, M. scutellaris, M. seminigra, T. angustula*	n.s.	Brazil	32.15–2968.54 mg GAE/100g	n.d.	176.07–5847.61 mg AAE/g o 258.24–8582.47 mg TE/100g DPPH both	Ethanol and methanol	n.d.	[147]
4	n.s.	n.s.	Portugal	n.d.	n.d.	14.41–25.24 ug/mL EC50 DPPH 161.73–251.83 ug/mL EC50 SOD 118.87–158.14 ug/mL EC50 Fe^2+^chel	Ethanol	UPLC-DAD-ESI/MS	[148]
1	n.s.	n.s.	India	269.1 and 159.1 mg GAE/g	25.50 and 57.25 mg QE/g	0.05 and 0.07 mg/mL EC50 DPPH	Ethanol (70%) and water	n.d.	[149]
n.s.	n.s.	n.s.	Portugal	5.28–6.27 mg Pinocembrin/mL	1.27–1.30 mg QE/mL	0.019–0.020 mg/mL EC50 ABTS 0.027–0.031 mg/mL EC50 DPPH 0.034–0.034 mg/mL EC50 SOD 39.5–49.9% Fe^2+^chel	Methanol, ethanol (70%) and water	n.d.	[150]
3	n.s.	n.s.	Algeria	15.84–61.63 mg GAE/100g	124.76–4946.53 mg CE/100	n.d.	Water, 50% ethanol, 85% ethanol, and 50%methanol	n.d.	[55]
11	n.s.	n.s.	Turkey	2748–19970 mg GAE/100g	3073–29175.0 mg QE/100g	1370.6–6332.9 mg TE/100g DPPH 2461.6–8580.3 mg TE/100g CUPRAC	Ethanol (70%)	LC-MS/MS	[151]
5	n.s.	n.s.	Serbia	1.45–5.31 g GAE/100mL	n.d.	0.093–0.346% EC50 DPPH	Ethanol	n.d.	[152]
48	n.s.	Poplar “orange”, “blue” and “third type”	Turkey	486.9 mg GAE/g orange 310.6 mg GAE/g blue 115.7 mg GAE/g third	265.7 mg QE/g orange 185.5 mg QE/g blue 109.53 mg QE/g third	65.64 %DPPH orange 42.22 %DPPH blue 26.49 %DPPH third	Ethanol (80%)	UHPLC–LTQ/orbitrap/MS/MS	[153]
1	n.s.	n.s.	India	5.15–20.99 mg GAE/g	8.39–14.26 mg QE/g	n.d.	Ethanol	HPTLC	[154]
9	*A. mellifera*, palynological identification	n.s.	Portugal	18.52–277.17 mg GAE/mL	6.34–142.32 mg CE/mL	n.d.	Water, methanol:water (80%) and ethanol:water (80%)	UV-VIS	[155]
5	n.s.	n.s.	Iraq	700–9333 µg CAE/mL	n.d.	40.0–83.3% DPPH	Methanol	HPLC–ESI/MS	[156]
1	*T. itama*	n.s.	Malaysia	n.d.	n.d.	90.7–99.34 % DPPH	Subsequent extractions: hexane, ethyl acetate and methanol	UV-VIS	[157]

TPC: total phenolics compounds; TFC: total flavonoids compounds; AOA: antioxidant activity; n.s.: not specified; n.d.: not determined; ^1^ A: Apis; H.: Heterotrigona, M.: Melipona, T.: Trigona; ^2^ GAE: gallic acid equivalents; CAE: caffeic acid equivalents; ^3^ QE: quercetin equivalents, CE: chrysin equivalents; ^4^ AAE: ascorbic acid equivalents; TE: Trolox equivalents, Fe^2+^: FeSO_4_*7H_2_0 equivalents.

**Table 5:** Summary of the results from studies on antioxidant capacity in beeswax, royal jelly and venom.

Sample Size	Botanical Origin/Bee Species ^1^	Sample Type	Country	TPC ^2^	TFC ^3^	AOA ^4^	Extraction	Characterization	References
**Beeswax**									
1	*A. mellifera*	Hydro-ethanolic extracts of honeycomb	China	1.62 mg GAE/g	1.62 mg/g (equivalent n.s.)	5.91 mg/ml EC50 DPPH 1.33 mg TE/g FRAP 0.38 mg Na_2_EDTA/g Fe^2+^chel.	Ethanol 75%	GC–MS	[161]
10	*A. mellifera*	Waste sediment separated from wax (5 MUD1 and 5 MUD2)	n.s.	1435.66 and 432.66 mg GAE/100g	295.84 and 142.17 mg CE/100g	1.60 and 0.23 mM TE TEAC 1.93–0.59 mM TE FRAP	Sediment with inorganic and organic waste was separated from wax honeycombs during recycling process following a heating process by steam (MUD1); the remaining wax was passed to a continuous decanter, where a fine sediment was generated (MUD2).	UPLC-DAD/ESI-MS	[20,162]
**Royal jelly**									
1	*A. mellifera*	Recombinant MRJPs 1–7	South Korea	n.d.	n.d.	DPPH (about 30–80%-graphicated)	n.s.	n.d.	[29]
28	*A. mellifera*	19 local and 9 commercial RJ	Romania	23.49 and 23.25 mg GAE/g	n.d.	37.23 and 35.94% DPPH 2.20 and 1.83 mM Fe^2+^/g FRAP	Water 10% (w/v)	n.d.	[163]
**Venom**									
1	*A. mellifera syriaca*	Venom	Lebanon	n.d.	n.d.	50–86.6% DPPH (from 2.5 to 500 µg/mL)	Lyophilized crude venom dissolved in 1 mL water (5 mg/mL)	LC-ESI-MS	[164]
5	*A. mellifera iberiensis*	Venom	Portugal	n.d.	n.d.	346–512 µg/mL EC50 DPPH 238–326 µg/mL EC50 RP 435–826 µg/mL EC50 BCB	Water (mg/mL)	LC/DAD/ESI-MS	[165]
4	*A. mellifera, A. cerana, A. florea, A. dorsata*	Venom	Thailand	n.d.	n.d.	DPPH, FRAP and ABTS (graphicated)	Various concentrations in PBS	HPLC-UV	[166]

TPC: total phenolic compounds; TFC: total flavonoid compounds; AOA: antioxidant activity; n.s.: not specified; n.d.: not determined; ^1^ A.: Apis; ^2^ GAE: gallic acid equivalents; ^3^ CE: chrysin equivalents; ^4^ TE: Trolox equivalents; Fe^2+^: FeSO_4_*7H_2_0 equivalents.

**Table 6:** In vitro antioxidant properties of bee products.

Bee Product	Bees Species ^1^	Cell culture/Substrate	Antioxidant Activity	Measurement	References
**Honey**					
Monofloral honeys (Italy)	*A. mellifera*	Bovine brain microsomes	Peroxyl-radical scavenging capacity	Time-course of TBA-RS formation during microsomal oxidation	[74]
Commercial multifloral honey (Italy)	*A. mellifera*	Human endothelial cell line (EA.hy926)	Cell membrane oxidation, intracellular oxidative damage, cell viability using MTT [3-(4,5-dimethyl-2-thiazolyl) -2,5-diphenyl -2H- tetrazolium bromide] assay and GSH analysis	Cytoprotective activity by fluorimetric determination, cell viability (the absorbance is proportional to the number of living cells) and microscopic evaluation	[16]
Buckwheat and Manuka honeys	n.s.	HepG2 cell lines, Cell Bank of Institute of the Biochemistry and Cell Biology, Chinese Academy of Sciences, Shanghai, China	Cellular antioxidant activity (CAA) and cytotoxicity assay	Peroxyl radical-induced oxidation of DCFH to DCF by fluorimetric determination and inhibition of oxidation by honey extracts (microscopic evaluation)	[17]
Malaysian kelulut honey	*Trigona* spp.	Lymphoblastoid cell line (LCL), AGRE, Los Angeles, CA, USA	Ferric-reducing antioxidant potential assay, total phenolic, and flavonoid content by UV spectrophotometry. Cell viability using MTS assay	Cell viability (%) reading the absorbance at 490 nm and positively affected by antioxidant properties	[18]
Monofloral honeys (China)	*A. dorsata*	HepG2 cell lines, Stem Cell Bank of Chinese Academy of Sciences	Cellular antioxidant activity (CAA) assay	Effective reduction of intracellular oxidative state reacting with peroxyl radicals or ROS/RNS. Fluorimetric determination	[19]
**Beeswax**					
Beeswax recycling by-product (MUD1)	*A. mellifera*	HepG2 cells, Biological Research Laboratory of Sevilla University, Spain	ROS concentration using CellROX^®^ Orange Reagent applied according to manufacturer’s instructions. Cells were analyzed with the Tali^®^ Image-Based cytometer	Intracellular ROS: percentage of cells with increased ROS levels related to the control	[20]
Two beeswax recycling by-products (MUD1 and MUD2)	*A. mellifera*	Adult skin HDF, GIBCO^®^ Invitrogen cell, Waltham, MA, USA	ROS concentration using CellROX^®^ Orange Reagent applied according to manufacturer’s instructions	Intracellular ROS: percentage of cells with increased ROS levels related to the control	[161]
**Pollen**					
Bee pollen (China)	n.s.	Blood from male Kunming mice	Superoxide dismutase (SOD) assay, lipid peroxidation index assay and total antioxidant capacity (T-AOC) assay	Spectrophotometric measurement of SOD content (U/mL), MDA content (nmol/mL) and inhibition rate (%)	[30]
Bee pollen from Jara pringosa (*Sistus ladanifer*) and Jara blanca (*Cistus albidus*) (Spain)	*A. mellifera*	Retinal ganglion cells (RGC-5, a rat ganglion cell-line transformed using E1A virus)	Antioxidant-capacity assay-measured the radicals induced in RGC-5 by the application of ROS (H_2_O_2_, O_2_^•−^, and HO)	Intracellular ROS: time-kinetic and concentration-response data for bee pollen towards production of various ROS in term of fluorescence intensity	[22]
Commercial pollens of different floral sources and geographical origins	n.s.	Livers obtained from pigs and homogenized	Inhibition of lipid peroxidation using thiobarbituric acid reactive substances (TBARS)	Spectrophotometric determination of inhibition ratio (%) and EC50 calculated (0.35–3.70 TBARS mg/mg extract)	[118]
**Bee bread**					
Beebread (Poland)	n.s.	Human glioblastoma cell line U87MG (HTB-14), ATCC, Rockville, MD, USA	Cytotoxicity evaluated by MTT assay. Total antioxidative ability related to phenolic and non-phenolic compounds after 24 h	Viability of U87MG (% of the control) after incubation with beebread, measuring the absorbance at 570 nm	[21]
**Propolis**					
Propolis	n.s.	Human erythrocytes from peripheral blood	Estimation of the inhibitory efficiency of propolis extracts on H_2_O_2_-induced lipid peroxidation using thiobarbituric acid (TBA) assay and protective effect of propolis extracts on H_2_O_2_-induced oxidative hemolysis	Measured the absorbance of the supernatant at 532 nm and calculated the hemolysis percentage	[47]
Propolis (Brazil)	*M. orbignyi*	Human erythrocytes from peripheral blood	Oxidative hemolysis inhibition assay, inhibitory efficiency against lipid peroxidation, cytotoxic activity and cell death profile (analysis performed using propidum iodide and annexin V-FITC dual staining)	Hemolysis (%), MDA (nmol/mL) and cell viability (%), respectively, spectrophotometrically determined and flow cytometric evaluation of death profile	[32]
Propolis (Portugal)	n.s.	Eukaryote unicellular model organism *S. cerevisiae* and human reconstituted skin tissue model (EpiDermTM EPI-200)	Evaluation of propolis protective effects against H_2_O_2_-induced oxidative stress and its influence on ROS intracellular levels in *S. cerevisiae* cells. UVB-induced overexpression of matrix metalloproteinases (MMPs), quantitative real-time PCR and immunohistochemistry (IHC) in skin tissue model	Viability and intracellular oxidation of *S. cerevisiae* cells analyzed for fluorescence by flow cytometry. Evaluation of the UVB-induced photoaging by immunohistochemistry and quantification of mRNA levels of MMPs	[142]
Propolis (Greece)	n.s.	Human immortalized keratinocyte (HaCaT) cell line, ATCC, Rockville, MD, USA	Determination of antioxidant capacity in cell lysates and assessment of protein oxidation by measuring the protein carbonyl colorimetric assay	DNA damage (AU) using fluorescence microscope, total antioxidant content and protein carbonyl content, spectrophotometrically determined	[23]
Propolis (Thailand)	n.s.	A549 human lung epithelial cells and HeLa cervical cancer cells	Determination of antioxidant activity by DPPH method and cytotoxicity by MTT assay	Extraction-method dependent antioxidant and flavonoid compounds. Cell shrinkage and floating in medium. Percentage of viability compared to the cell control	[24]
Propolis (Turkey)	n.s.	Human foreskin fibroblast cells (CRL-2522), ATCC, Manassas, VA, USA	Spectrofluorometric analysis of intracellular oxidative stress with CM-H_2_DCFDA	ROS levels measured by spectrofluorometric method	[25]
Brazilian green propolis from *Baccharis dracunculifolia* (Minas Gerais State, Brazil)	*A. mellifera*	Retinal ganglion cells (RGC-5, a rat ganglion cell-line transformed using E1A virus)	Antioxidant-capacity assay measured the radicals induced in RGC-5 by the application of ROS (H2O2, O2·-, and HO)	Intracellular ROS: time-kinetic and concentration-response data for propolis towards production of various ROS in terms of fluorescence intensity	[22]
Red propolis (Brazil)	n.s.	Human tumor cell lines HL-60 (leukemia), PC3 (prostate carcinoma), SNB19 (glioblastoma), and HCT-116 (colon carcinoma), National Cancer Institute, USA	High in vitro antioxidant activity related to total phenolic and flavonoid compound content. MTT assay to determine the cytotoxic (antitumor) potential of the extracts	Growth inhibition of tumor cell lines (%), using spectrophotometer	[26]
Propolis (Cameroon)	n.s.	Diluted human whole blood, mouse macrophage cell line J774.2, European Collection of Cell Cultures (UK) and NIH-3 T3 fibroblast cells, ATCC, Manassas, USA	Oxidative burst assay (luminol-enhanced chemiluminescence assay), nitric oxide assay and MTT cytotoxicity assay	ROS inhibition (EC50 µg/mL), NO inhibition (EC50 µg/mL) and cytotoxicity (EC50 µg/mL), respectively, using spectrophotometer	[33]
Propolis (Morocco)	n.s.	Human monocytic cell line THP-1 (ATCC 202-TIB), human colorectal carcinoma cell line HCT-116 (ATCC^®^ CCL-247™) and breast cancer cell line MCF-7 (ATCC^®^HTB-22™)	High antioxidant content and activity by scavenging free radicals with IC50 (DPPH = 0.02, ABTS = 0.04, and FRAP = 0.04 mg/ml). MTT assay for cytotoxic and cytostatic activity and cell viability determination	Total phenols, flavone, and flavonol and antioxidant activity affect cell viability defined as the ratio (%) of absorbance of treated cells to untreated cells (control)	[27]
Propolis (Poland)	n.s.	Fresh human erythrocyte concentrates (65%), Blood bank in Poznan, Poland	High antioxidant potential related to DPPH free-radical scavenging activity and reducing power; significant protection of human red blood cells from oxidative damage. Hemolysis assays	Hemolysis (%) estimated by measuring absorbance of the supernatant; microscope studies of erythrocyte shape transformation (Bessis classification) and inhibition of free-radical-induced hemolysis	[34]
**Royal jelly**					
Enzyme-treated royal jelly (Jiangshan, China)	*A. mellifera*	Peritoneal macrophages, BALB/c mice	Cell viability MTT assay and ROS, SOD and GSH quantification according to the manufacturer’s kit instructions	Intracellular ROS and NO production; activity of the enzyme SOD and concentration of the antioxidant GSH (spectrophotometric quantification)	[180]
Fresh royal jelly from Yangtze Valley, People’s Republic of China	*A. mellifera*	Retinal ganglion cells (RGC-5, a rat ganglion cell-line transformed using E1A virus)	Antioxidant-capacity assay measured the radicals induced in RGC-5 by the application of ROS (H_2_O_2_, O_2_·^-^, and HO)	Intracellular ROS: time-kinetic and concentration-response data for royal jelly towards production of various ROS in terms of fluorescence intensity	[22]
Fresh royal jelly (Korea) and recombinant AcMRJP2 protein	*A. cerana*	Murine fibroblast cell line NIH 3 T3	Antioxidant activity and shielding of the cell against oxidative stress and DNA protection against ROS. Cell viability measured by MTT assay, apoptosis assay and DNA protection assay	Antioxidant activity determines increased cell viability (%), reduced caspase-3 activity and apoptosis in the cells using laser-scanning confocal microscopy. DNA nicking assay in a metal-catalyzed oxidation system observed by agarose gel electrophoresis	[28]
Recombinant AmMRJPs 1–7	*A. mellifera*	Murine fibroblast cell line NIH 3 T3	Radical scavenging activity and protection against DNA oxidative damage. Cell viability measured by MTT assay, apoptosis assay and DNA protection assay	Antioxidant activity determines increased cell viability (%), reduced caspase-3 activity and apoptosis in the cells using laser-scanning confocal microscopy. DNA nicking assay in a metal-catalyzed oxidation system observed by agarose gel electrophoresis	[29]
**Bee Venom**					
Melittin (Northeast Portugal)	*A. mellifera iberiensis*	MCF-7, NCI-H460, HeLa and HepG2 tumour lines	Free-radical scavenging activity, reducing power, lipid peroxidation inhibition and high capacity to inhibit NO production.	Chemical characterization by LC/DAD/ESI-MS; DPPH for free-radical scavenging activity; reducing power measuring the absorbance at 690 nm	[165]

^1^ A: Apis; M.: Melipona; n.s.: not specified.
